# The standardised copy of pentagons test

**DOI:** 10.1186/1744-859X-10-13

**Published:** 2011-04-11

**Authors:** Konstantinos N Fountoulakis, Melina Siamouli, Panagiotis T Panagiotidis, Stamatia Magiria, Sotiris Kantartzis, Vassiliki A Terzoglou, Timucin Oral

**Affiliations:** 1 Third Department of Psychiatry, School of Medicine, Aristotle University of Thessaloniki, Thessaloniki, Greece; 2 Asklipios Clinic, Veroia, Greece; 3 424 General Military Hospital of Thessaloniki, Thessaloniki, Greece; 4 School of Medicine, Aristotle University of Thessaloniki, Thessaloniki, Greece; 5 Psychologist, Thessaloniki, Greece; 6 Fifth Inpatient Department of Psychiatry and Outpatient Unit of Mood Disorders, Bakirköy State Teaching and Research Hospital for Neuropsychiatry, Istanbul, Turkey

## Abstract

**Background:**

The 'double-diamond copy' task is a simple paper and pencil test part of the Bender-Gestalt Test and the Mini Mental State Examination (MMSE). Although it is a widely used test, its method of scoring is crude and its psychometric properties are not adequately known. The aim of the present study was to develop a sensitive and reliable method of administration and scoring.

**Methods:**

The study sample included 93 normal control subjects (53 women and 40 men) aged 35.87 ± 12.62 and 127 patients suffering from schizophrenia (54 women and 73 men) aged 34.07 ± 9.83.

**Results:**

The scoring method was based on the frequencies of responses of healthy controls and proved to be relatively reliable with Cronbach's α equal to 0.61, test-retest correlation coefficient equal to 0.41 and inter-rater reliability equal to 0.52. The factor analysis produced two indices and six subscales of the Standardised Copy of Pentagons Test (SCPT). The total score as well as most of the individual items and subscales distinguished between controls and patients. The discriminant function correctly classified 63.44% of controls and 75.59% of patients.

**Discussion:**

The SCPT seems to be a satisfactory, reliable and valid instrument, which is easy to administer, suitable for use in non-organic psychiatric patients and demands minimal time. Further research is necessary to test its psychometric properties and its usefulness and applications as a neuropsychological test.

## Background

The 'double-diamond copy' task is a well known, simple paper and pencil test included in the Bender-Gestalt Test [1-9]. A slightly different version ('double-pentagon copy') with a different overlapping shape is included also in the Mini Mental State Examination (MMSE) [10,11]. It is composed of two overlapping pentagons, with the overlapping shape being a rhombus. It assesses visual motor ability. However, for both scales this item is scored in a very simple way. For example, in the MMSE it receives a 0/1 score and in the Bender-Gestalt Test a 0-4 score, with sample drawings to lead the examiner. The overall method is more 'qualitative' and focuses on the 'organic/neuropsychiatric' end of the spectrum (for example, dementia), since scoring levels 0-2 are reserved for very poor performance.

**Figure 1:**
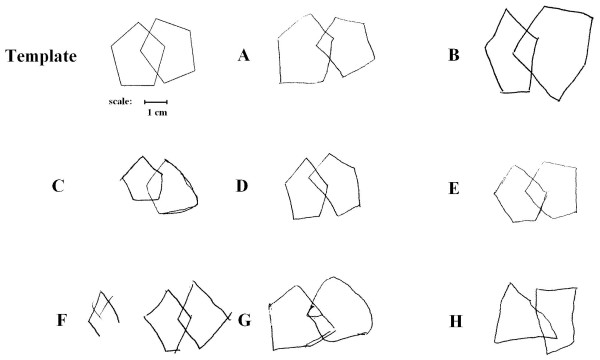
**Template and samples showing how patients with schizophrenia perform in the copy of pentagons task**.

Non-organic psychiatric patients, however, including most patients with schizophrenia, are likely to receive a score of 2-4. Samples showing how patients with schizophrenia perform in this task are shown in Figure 1. It is obvious that by using these scoring methods to assess the drawings of psychiatric patients, valuable information might be lost.

The aim of the current study was to develop a novel and detailed standardised method for the administration and scoring of a task similar to the 'double-diamond copy' task. This task included two pentagons overlapping into a rhombus but with a slightly different shape in comparison to the Bender-Gestalt figure (Figure 1). This new task with his novel scoring method aims to be reliable, valid and sensitive to change in response to treatment and be suitable for use in mental patients suffering from other disorders than dementia.

## Methods

### Study sample

The study sample included 93 normal control subjects (53 women (56.98%) and 40 men (43.02%)) aged 35.87 ± 12.62 (range 18-68) and 127 patients suffering from schizophrenia, undifferentiated type, according to the *Diagnostic and Statistical Manual of Mental Disorders*, fourth edition, text revision (DSM-IV-TR) (54 women (42.52%) and 73 men (57.48%)) aged 34.07 ± 9.83 (range 18-66).

All subjects were physically healthy with normal clinical and laboratory findings. All control subjects and patients gave informed consent and the protocol received approval by the University's Ethics Committee. The patients were either inpatients or outpatients of a private psychiatric clinic.

### Clinical diagnosis

The diagnosis was made according to DSM-IV-TR criteria on the basis of a semistructured interview based on the Schedules for Clinical Assessment in Neuropsychiatry version 2.0 (SCAN v 2.0) [12].

Normal controls were assessed on the basis of an unstructured clinical interview.

### The Standardised Copy of the Pentagons Test (SCPT) procedure

The SCPT procedure demanded the subject to copy a shape of two partially overlapping pentagons analogous to a shape of the Bender-Gestalt Test and similar to the figure used in some versions of the MMSE. The shape includes two pentagons whose overlap is a four-angle rhombus. The shape is shown in Figure 1 and in Additional file 1. The SCPT instructions ask the subject to draw an identical shape on the same piece of paper. The template shape was printed on the left half of the sheet leaving space for the subject to reproduce it on the right. No time limit was set and no time recording was made.

The assessment included the Random Letter Test (RLT) for the assessment of attention and vigilance [13]. It includes the following four series of letters: LTPEAOAISTDALAA; ANIABFSAMPZEOAD; PAKLATSXTOEABAA and ZYFMTSAHEOAAPAT. The first and third group include five 'A's, while the second and the fourth include four 'A's. The test requires the patient to hit the desk when the examiner pronounces 'A'. Errors of omission and commission are recorded. It is expected (and verified in the present study) that the mean number of errors expected from normal controls in this test is around 0.2 [14]. Both errors of omission and commission were registered for this test.

### Psychometric assessment

The psychometric assessment included the Positive and Negative Symptoms Scale (PANSS) [15], the Young Mania Rating Scale (YMRS) [16], and the Montgomery Asberg Depression Rating Scale (MADRS) [17].

### Statistical analysis

Frequency tables were created concerning the scores of healthy controls. These tables were used to produce percentile scores and develop a scoring method for the scale. The Pearson's R correlation coefficient, factor analysis (varimax normalised rotation) and item analysis [18] (calculation of Cronbach's α) were used to explore the internal structure of the scale. Analysis of variance (ANOVA) [19], was used to test the difference between groups, and was performed separately for subjects below and above the age of 40. Discriminant function analysis was also used to explore the power of the scale in discriminating between groups. The Pearson's R correlation coefficient was calculated to assess the test-retest reliability as well as the inter-rater reliability. However, the calculation of correlation coefficients is not a sufficient method to test reliability and reproducibility of a method and its results, because it is an index of correlation and not an index of agreement [19-21]. The calculation of means and standard deviations for each SCPT item and total score during the first (test) and second (retest) applications may provide an impression of the stability of results over time.

The means and the standard deviations of the differences concerning each SCPT item between test and retest were also calculated, and plots of the test vs retest and difference vs average value for each variable were generated. In fact, it is not possible to use statistics to define acceptable agreement [19]. However, these plots may assist decision. This method has been used in previous studies concerning the validation of scientific methods [22,23].

## Results

The frequency tables for scores of healthy controls are shown in Table 1. In the same table, the proposed scoring for each item is also shown. This scoring method is based on the frequencies of responses of healthy controls (percentile scores).

**Table 1:** Frequencies of normal control results for each item, and proposed standardised score on the basis of percentiles

Raw score	No. of observations	Percentage of observations	Standard score
Number of 'A' omissions			

0	92	98.92	100

1	1	1.08	0

> 1	0	0.00	0

Total	93	100.00	

Number of 'A' intrusions			

0	86	92.47	100

1	6	6.45	8

2	1	1.08	1

> 2	0	0.00	0

Total	93	100.00	

1. Number of left pentagon angles missing (maximum 5)			

0	93	100.00	100

> 0	0	0.00	0

Total	93	100.00	

2. Number of right pentagon angles missing (maximum 5)			

0	92	98.92	100

> 0	1	1.08	1

Total	93	100.00	

3. Number of angles of the overlapping shape (rhombus) missing or in excess			

0	92	98.92	100

> 0	1	1.08	1

Total	93	100.00	

4. Numbers of breaks and corrections in the lines of the two pentagons			

0	22	23.66	100

1	36	38.71	75

2	18	19.35	35

3	3	3.23	20

4	6	6.45	15

5	7	7.53	10

> 5	1	1.08	1

Total	93	100.00	

5. Severe distortion in the proportions in the left pentagon shape			

0	73	78.49	100

> 0	20	21.51	20

Total	93	100.00	

6. Severe distortion in the proportions in the right pentagon shape			

0	67	72.04	100

> 0	26	27.96	30

Total	93	100.00	

7. Severe distortion of the proportions of the rhombus shape			

0	60	64.52	100

> 0	33	35.48	35

Total	93	100.00	

8. Angles with a reverse orientation			

0	89	95.70	100

> 0	4	4.30	5

Total	93	100.00	

9. Asymmetry of pentagons			

0	79	84.95	100

> 0	14	15.05	15

Total	93	100.00	

10. Smaller size in comparison to the template			

0	72	77.42	100

> 0	21	22.58	20

Total	93	100.00	

11. Sides not straight lines			

0	38	40.86	100

1	24	25.81	60

2	22	23.66	35

3	8	8.60	10

> 3	1	1.08	1

Total	93	100.00	

12. Angles whose sides are not straight lines			

0	67	72.04	100

1	12	12.90	30

2	8	8.60	15

3	5	5.38	6

> 3	1	1.08	1

Total	93	100.00	

13. Rotation			

No	90	96.77	100

Yes	3	3.23	3

Total	93	100.00	

14. Crossing sides			

0	93	100.00	100

> 0	0	0.00	0

Total	93	100.00	

15. Close-in			

0	93	100.00	100

> 0	0	0.00	0

Total	93	100.00	

The one-way ANOVA revealed significant difference in the total SCPT score in comparison to controls for subjects under the age of 40 (*P *< 0.001) but not for those above this age (*P *= 0.17; Table 2). Note that SCPT-14 and SCPT-15 had no variance so they were not included in the analysis concerning separate items. The results are shown in Table 2 along with post hoc tests. It seems that in older subjects there are no differences because the performance of controls gets worse, while the change in the performance of patients is not great.

**Table 2:** Comparison of the scores of normal controls and schizophrenic patients (analysis of variance (ANOVA)) above and below 40 years of age, with t test as post hoc test

	Controls		Patients with schizophrenia		*P *value
		
	Mean	SD	Mean	SD	
Below 40 years					

RLT-A	100.00	0.00	71.43	45.72	< 0.001

RLT-B	84.14	21.31	65.00	40.05	< 0.001

SCPT-1	100.00	0.00	98.02	14.00	NS

SCPT-2	100.00	0.00	92.16	26.87	< 0.05

SCPT-3	98.29	13.00	93.14	25.27	NS

SCPT-4	59.17	32.80	57.93	33.02	NS

SCPT-5	84.83	31.64	66.73	39.63	< 0.01

SCPT-6	79.48	32.14	68.12	35.03	< 0.05

SCPT-7	77.59	31.17	61.39	32.08	< 0.01

SCPT-8	93.45	24.28	96.24	18.62	NS

SCPT-9	89.74	27.93	76.44	38.24	< 0.05

SCPT-10	84.83	31.64	86.53	30.08	NS

SCPT-11	64.67	31.74	47.86	33.32	< 0.01

SCPT-12	80.83	34.64	47.26	41.35	< 0.001

SCPT-13	94.98	21.67	93.28	24.76	NS

SCPT-14	100.00	0.00	100.00	0.00	NS

SCPT-15	100.00	0.00	100.00	0.00	NS

SCPT	1307.86	140.59	1185.09	161.50	< 0.001

Above 40 years					

RLT-A	96.77	17.96	84.62	37.55	NS

RLT-B	87.13	15.98	62.46	43.00	< 0.01

SCPT-1	100.00	0.00	96.67	18.26	NS

SCPT-2	97.25	16.50	100.00	0.00	NS

SCPT-3	100.00	0.00	96.70	18.07	NS

SCPT-4	64.44	31.12	53.73	38.83	NS

SCPT-5	77.78	36.34	60.00	40.68	NS

SCPT-6	80.56	31.80	74.33	34.31	NS

SCPT-7	74.72	32.14	69.67	32.98	NS

SCPT-8	97.36	15.83	96.83	17.34	NS

SCPT-9	81.11	35.84	91.50	25.94	NS

SCPT-10	75.56	37.37	84.00	32.55	NS

SCPT-11	65.03	34.61	45.73	34.34	< 0.05

SCPT-12	70.11	40.89	56.30	42.25	NS

SCPT-13	97.31	16.17	87.07	33.54	NS

SCPT-14	100.00	0.00	100.00	0.00	NS

SCPT-15	100.00	0.00	100.00	0.00	NS

SCPT	1281.22	151.58	1212.53	121.71	< 0.05

For below 40 years there were 60 controls and 101 patients. For above 40 years there were 33 controls and 26 patients.

NS = not significant; RLT = Random Letter Test; SCPT = Standardised Copy of Pentagons Test.

The Pearson's R correlation coefficients for the SCPT items are shown in Table 3 (total study sample).

**Table 3:** Pearson Correlation coefficients (R) among the Standardised Copy of Pentagons Test (SCPT) items and Random Letter Test (RLT) scores in the total study sample

	SCPT-1	SCPT-2	SCPT-3	SCPT-4	SCPT-5	SCPT-6	SCPT-7	SCPT-8	SCPT-9	SCPT-10	SCPT-11	SCPT-12	SCPT-13	SCPT-14	SCPT-15
RLT-A															

RLT-B	**0.48**														

SCPT-1	1.00														

SCPT-2	**0.17**	1.00													

SCPT-3	**0.37**	**0.19**	1.00												

SCPT-4	0.02	0.03	0.01	1.00											

SCPT-5	0.00	**0.14**	**0.14**	0.03	1.00										

SCPT-6	0.07	0.12	**0.26**	0.12	**0.28**	1.00									

SCPT-7	-0.03	0.08	0.12	0.02	**0.33**	**0.49**	1.00								

SCPT-8	-0.02	-0.04	0.07	0.04	0.02	0.10	0.01	1.00							

SCPT-9	0.04	0.07	0.07	0.11	**0.19**	**0.22**	**0.19**	0.00	1.00						

SCPT-10	-0.06	0.12	0.01	0.07	-0.04	0.07	0.04	0.05	0.05	1.00					

SCPT-11	0.05	0.05	0.02	0.07	**0.25**	**0.19**	0.12	0.07	0.04	0.06	1.00				

SCPT-12	**0.13**	0.06	-0.01	-0.03	**0.19**	0.05	0.00	-0.02	0.09	0.04	**0.35**	1.00			

SCPT-13	-0.03	0.03	0.12	0.00	0.05	**0.18**	**0.18**	**0.19**	-0.05	0.12	0.06	0.11	1.00		

SCPT-14														1.00	

SCPT-15															1.00

SCPT	**0.19**	**0.31**	**0.34**	**0.31**	**0.56**	**0.62**	**0.53**	**0.21**	**0.43**	**0.30**	**0.50**	**0.45**	**0.34**		

Values significant at *P *< 0.05 are shown in bold.

The Pearson's R correlation coefficients for the SCPT items and the Positive and Negative Syndrome Scale (PANNS; positive, negative and general psychopathology subscales), the YMRS and the MADRS are shown in Table 4 (only for patients with schizophrenia).

**Table 4:** Pearson Correlation coefficients (R) among the Standardised Copy of Pentagons Test (SCPT) items and subscales and the psychometric scales scores in schizophrenic patients only

	PANSS-Positive	PANSS-Negative	PANSS-General psychopathology	YMRS	MADRS
RLT-A	0.00	0.06	0.08	-0.14	-0.11

RLT-B	-0.02	-0.03	-0.04	0.07	**-0.16**

SCPT-1	0.01	-0.10	-0.02	0.01	**-0.18**

SCPT-2	**-0.15**	**-0.19**	**-0.17**	-0.02	-0.06

SCPT-3	-0.03	-0.16	-0.14	0.03	**-0.33**

SCPT-4	-0.05	-0.02	-0.04	**-0.23**	-0.02

SCPT-5	**-0.27**	**-0.27**	**-0.28**	**-0.17**	**-0.24**

SCPT-6	**-0.17**	**-0.29**	**-0.25**	**-0.17**	**-0.16**

SCPT-7	-0.12	**-0.24**	**-0.17**	-0.09	**-0.17**

SCPT-8	0.09	0.07	0.11	-0.06	0.06

SCPT-9	0.04	-0.06	-0.06	0.09	-0.02

SCPT-10	0.08	0.04	0.09	-0.04	-0.02

SCPT-11	-0.12	**-0.29**	**-0.22**	-0.03	**-0.21**

SCPT-12	**-0.24**	**-0.40**	**-0.34**	-0.10	**-0.29**

SCPT-13	-0.14	**-0.20**	-0.12	0.01	0.06

SCPT-14	-	-	-	-	-

SCPT-15	-	-	-	-	-

SCPT total	**-0.21**	**-0.39**	**-0.31**	**-0.15**	**-0.29**

Deficit index (DcI)	-0.05	**-0.16**	-0.10	-0.02	**-0.18**

Missing angles (MA)	-0.08	**-0.21**	**-0.16**	0.01	**-0.27**

Size (S)	0.00	-0.06	0.00	-0.04	-0.04

Deformation index (DfI)	**-0.21**	**-0.37**	**-0.33**	-0.14	**-0.26**

Proportion (P)	**-0.19**	**-0.31**	**-0.28**	-0.12	**-0.22**

Quality of lines (QL)	**-0.22**	**-0.41**	**-0.34**	-0.08	**-0.30**

Correction (C)	-0.01	-0.06	-0.06	-0.09	-0.03

Image distortion (ID)	-0.03	-0.09	-0.01	-0.03	0.08

Close-in index (CiI)	**-0.22**	**-0.41**	**-0.34**	-0.08	**-0.30**

Quality of lines (QL)	**-0.22**	**-0.41**	**-0.34**	-0.08	**-0.30**

Close-in (CI)	-	-	-	-	-

Values significant at *P *< 0.05 are shown in bold. Items 14 and 15 have no variance so a correlation coefficient cannot be calculated for them.

MADRS = Montgomery Asberg Depression Rating Scale; PANSS = Positive and Negative Symptoms Scale; RLT = Random Letter Test; YMRS = Young Mania Rating Scale.

The results of the factor analysis (varimax normalised rotation) are shown in Table 5. The analysis (by using the Keiser-Fleish criterion of eigenvalues larger than 1) produced six factors explaining 62% of the total variance. On the basis of this factor analysis, subscales were created and the differences between groups concerning these subscales are also shown in Table 6. The last SCPT item (closing-in) was included as a seventh subscale since it did not contribute to the factor analysis. One-way ANOVA revealed significant differences between the two diagnostic groups and post hoc tests showed that this difference concerned the some of the subscales but not all (*P *< 0.001; Table 6).

**Table 5:** Factor analysis of Standardised Copy of Pentagons Test (SCPT) items (varimax normalised rotation) of the whole sample

	Factor 1	Factor 2	Factor 3	Factor 4	Factor 5	Factor 6
SCPT-1	-0.11	**0.82**	0.14	-0.07	-0.05	-0.06

SCPT-2	0.14	**0.40**	0.05	-0.22	**0.57**	0.05

SCPT-3	0.23	**0.77**	-0.09	0.17	0.04	0.03

SCPT-4	-0.01	0.02	0.01	0.10	0.07	**-0.86**

SCPT-5	**0.61**	0.04	0.37	-0.09	-0.09	0.02

SCPT-6	**0.73**	0.18	0.03	0.20	0.07	-0.16

SCPT-7	**0.82**	-0.05	-0.05	0.08	0.05	0.06

SCPT-8	-0.02	0.03	0.02	**0.75**	-0.12	-0.20

SCPT-9	**0.43**	0.02	0.05	-0.26	0.05	**-0.45**

SCPT-10	-0.03	-0.15	0.03	0.15	**0.84**	-0.12

SCPT-11	0.15	-0.02	**0.76**	0.11	0.00	-0.10

SCPT-12	-0.02	0.07	**0.83**	-0.03	0.08	0.07

SCPT-13	0.19	0.02	0.07	**0.67**	0.24	0.24

Percentage of total	15%	11%	11%	10%	9%	8%

Total variance explained						64%

Values significant at *P *< 0.05 are shown in bold.

**Table 6:** comparison between the two diagnostic groups (one-way ANOVA) concerning SCPT subscales comparison between the two diagnostic groups (one-way ANOVA) concerning SCPT subscales

	Normal controls		Patients with schizophrenia		*P *value
	
	Mean	SD	Mean	SD	
Deficit index (DcI)	478.12	43.56	465.96	72.46	< 0.001

Missing angles (MA)	297.89	14.36	286.04	43.97	0.01

Size (S)	180.22	37.34	179.91	40.98	NS

Deformation index (DfI)	909.11	135.16	808.67	146.86	NS

Proportion (P)	324.95	86.49	279.44	95.27	< 0.001

Quality of lines (QL)	141.53	55.94	97.24	60.37	< 0.001

Correction (C)	147.63	47.46	136.99	51.65	NS

Image distortion (ID)	290.82	34.66	287.86	34.18	NS

Close-in index (CiI)	241.53	55.94	197.24	60.37	NS

Quality of lines (QL)	141.53	55.94	97.24	60.37	< 0.001

Close-in (CI)	100.00	0.00	100.00	0.00	NS

The correlation coefficients for these subscales are shown in Table 7. Some correlations among these scales are statistically significant but weak. A second factor analysis of these subscales produced three superfactors explaining 22%, 22% and 15% of total variance, respectively. The first one included subscales 2 and 5, the second included subscales 1, 3, 4 and 6, and the third included subscales 3 and 7 (Table 8).

**Table 7:** Correlation coefficients among the Standardised Copy of Pentagons Test (SCPT) subscales

	P	MA	QL	ID	S	C
Proportion (P)						

Missing angles (MA)	0.28					

Quality of lines (QL)	0.24	0.16				

Image distortion (ID)	0.13	0.04	0.08			

Size (S)	0.18	0.56	0.11	0.08		

Correction (C)	0.45	0.18	0.10	0.04	0.14	

Close-in (CI)	0.01	0.06	0.06	-0.02	-0.03	-0.04

**Table 8:** Factor analysis of the subscales (second order factor analysis)

	Second-order factor 1	Second-order factor 2	Second-order factor 3
Factor 1	0.17	**0.81**	0.09

Factor 2	**0.86**	0.16	0.12

Factor 3	0.10	**0.41**	**0.49**

Factor 4	0.02	**0.30**	0.03

Factor 5	**0.89**	0.06	-0.05

Factor 6	0.08	**0.78**	-0.11

Factor 7	-0.01	-0.13	**0.89**

Explained variance	1.57	1.57	1.06

Proportion of variance explained	22%	22%	15%

Total variance explained	-	-	59%

Values significant at P < 0.05 are shown in bold.

Item analysis (calculation of Cronbach's α) Cronbach's α was equal to 0.61. The α coefficient did not change significantly when any item was omitted from the analysis.

The Discriminant Function Analysis results are shown in Tables 9 and 10. This analysis produced the following function: When 3 (SCPT-1) + 9 × (SCPT-2) + 10 × (SCPT-3) + 6 × (SCPT-4) + 4 × (SCPT-5) - 2 × (SCPT-6) + 12 × (SCPT-7) - 6 × (SCPT-8) + 1 × (SCPT-9) - 9 × (SCPT-10) + 9 × (SCPT-11) + 15 × (SCPT-12) + 4 × (SCPT-13) > 4456 then the subject is likely to be a normal control rather than a schizophrenic patient. This function correctly classified 63.44% of controls and 75.59% of patients with schizophrenia, which is a satisfactory performance.

**Table 9:** Discriminant function analysis results

Diagnosis	Percentage classified correct	Classified as normal controls	Classified as schizophrenic patients	Total
Normal controls	63.44	59	31	90

Schizophrenic patients	75.59	34	96	130

Total	70.45	93	127	220

**Table 10:** Discriminant function analysis coefficients

	Normal control function coefficients	Schizophrenic patient function coefficients	Difference of coefficients	Final function coefficient (difference × 1000)
Constant	-73.025	-68.569	-4.456	-4456

SCPT-1	0.732	0.729	0.003	3

SCPT-2	0.173	0.164	0.009	9

SCPT-3	0.046	0.036	0.01	10

SCPT-4	0.038	0.032	0.006	6

SCPT-5	0.026	0.022	0.004	4

SCPT-6	-0.05	-0.048	-0.002	-2

SCPT-7	0.065	0.053	0.012	12

SCPT-8	0.228	0.234	-0.006	-6

SCPT-9	0.052	0.051	0.001	1

SCPT-10	0.052	0.061	-0.009	-9

SCPT-11	0.024	0.015	0.009	9

SCPT-12	0.01	-0.005	0.015	15

SCPT-13	0.117	0.113	0.004	4

SCPT = Standardised Copy of Pentagons Test.

The Pearson's R correlation coefficient (R) for inter-rater reliability is 0.52 for the total SCPT scale and ranges from 0.46 to 0.86 for individual items (Table 11); with regard to test-retest reliability, the same coefficient was equal to 0.46 and the items coefficients ranged from -0.12 to 0.70 (Table 9). Retest was performed within 5 days of first testing. The calculation of means and standard deviations for each SCPT item and total score during the first (test) and second (retest) applications as well as the plots of the test vs retest and difference vs average value for each variable suggested that the SCPT is reliable and replicable.

**Table 11:** Inter-rater and test-retest reliability coefficients

Item	Inter-rater reliability	Test-retest reliability
SCPT-1	-	0.56

SCPT-2	0.81	-0.03

SCPT-3	0.55	-

SCPT-4	0.86	-0.02

SCPT-5	0.46	0.27

SCPT-6	0.61	0.51

SCPT-7	0.63	0.24

SCPT-8	0.48	-

SCPT-9	0.70	-0.12

SCPT-10	0.66	0.29

SCPT-11	0.71	0.70

SCPT-12	0.14	0.46

SCPT-13	0.48	-0.03

SCPT-14	-	-

SCPT-15	-	-

SCPT	0.52	0.46

Deficit index (DcI)	0.46	0.21

Missing angles (MA)	0.42	0.38

Size (S)	0.64	0.14

Deformation index (DfI)	0.66	0.33

Proportion (P)	0.62	0.39

Quality of lines (QL)	0.43	0.57

Correction (C)	0.81	-0.04

Image distortion (ID)	0.41	-0.03

Close-in index (CiI)	0.38	0.57

Close-in (CI)	-	-

SCPT = Standardised Copy of Pentagons Test.

## Discussion

The SCPT is a test of visual motor ability, and although several decades have passed since it was introduced, little has been performed to standardise it. This may be due to its complex pattern and a preference to score it on the basis of an 'overall' impression or 'qualitatively'. Little data can be found in the literature and these exist only because it is included in the MMSE and the Bender-Gestalt Test. Until now, scoring has been based on the overall impression and quality of the drawing as well as on common errors observed. The focus is on detecting 'organic' brain defects (for example, due to tumour, stroke or dementia), however, in this way many details in the performance of patients may be lost, and this is especially true when the test is used in psychiatric populations. Even the Bender-Gestalt Test uses a very simple way to score these tests.

**Figure 2:**
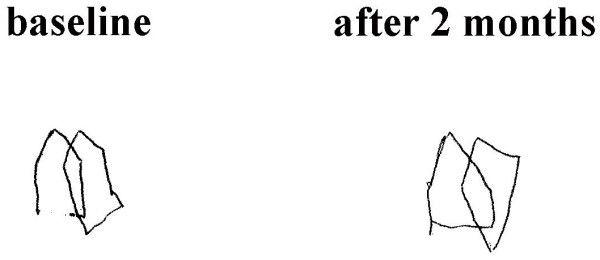
**Improvement in the performance in the copy of pentagons task in a patient after 2 months of antipsychotic treatment**.

The current study attempted to develop a standardised scoring method that would allow the examiner to reliably quantify the subject's performance in the copy the pentagons test. This test demands the subject to copy a simple drawing template. Both the drawing template and the resulting SCPT along with the scoring method developed by the current study are shown in Additional file 1. The test and its scoring method proved to be satisfactory reliable and stable. It is not clear whether it is also sensitive to change after treatment. In one patient, performance improved after 2 months of antipsychotic treatment (Figure 2). However, it is still necessary to apply the test to different patient populations, especially to patients suffering from 'organic' brain disease, before and after therapeutic intervention.

The scoring method is such that it allows for maximum contrast and differentiation between normal subjects and psychiatric patients. It also leaves little space for subjective assessment. In essence, the proposed scoring method expands levels 2-4 of the Bender-Gestalt scoring system.

Although some of the correlation coefficients among individual SCPT items were significant, overall each item assesses a distinct issue. This is also reflected in factor analysis. The six factors that emerge explain roughly 10% of the total variance each and 64% combined. The SCPT can be divided into subscales on the basis of the factor analysis and its interpretation. In this way, six subscales can be created. The first factor includes items 5, 6, 7 and 9 and largely reflects 'proportion'. Thus it may constitute the basis of a subscale named 'proportion' (P). The second one includes items 1, 2 and 3 and reflects the number of missing angles in the drawing. Thus it constitutes the basis of a subscale under the title 'missing angles' (MA). The third factor includes items 11 and 12 and reflects the quality of the line drawing in the shape. The resulting subscale is named 'quality of lines' (QL). The fourth factor includes items 8 and 13 (and 14, although that item's variance did not permit to include it in the factor analysis) and is an index of image distortion, and constitutes the basis of the 'image distortion' (ID) subscale. The fifth includes items 2 (again) and 10 and reflects differences in size between the template and the shape designed by the subject, thus being the basis of the 'size' (S) subscale. The sixth factor includes items 4 and 9 (again) and reflects correction efforts, giving rise to the 'correction' (C) subscale. A final subscale, which includes only item 15 and is named 'closing-in' (CI), should be added. Schizophrenic patients differ from controls in P, MA and QL but not concerning the rest subscales.

Correlations among these subscales are significant but weak. The factor analysis of these subscales produced three superfactors, named 'indices'. The first (subscales MA and S) constitutes the 'deficit index' (DcI), while the second (subscales P, QL and C) is the 'deformation index' (DfI). The third index (subscales QL and CI) is the 'closing-in index' (CiI). It is important to note that all the items of the SGST included in the DcI are easy for the normal subject, while the more difficult ones (2, 5 and 8) are included in the DfI. Patients differ from controls concerning DfI and CiI indices (*P *< 0.001) but not DcI. In the context of the above, the SCPT is divided into the following three indices and six subscales:

a. Deficit index (DcI), which includes the following two subscales:

1. Missing angles (ME) subscale (items 1, 2 and 3)

2. Size (S) subscale (items 2 and 10).

b. Deformation index (DfI), which includes the following three subscales:

1. Proportion (P) subscale (items 5, 6, 7 and 9)

2. Quality of lines (QL) subscale (items 11 and 12)

3. Corrections (C) subscale (items 4 and 9)

4. Image distortion (ID) subscale (items 8, 13 and 14).

c. Closing-in index (CiI), which includes the following two subscales:

1. Quality of lines (QL) subscale (items 11 and 12)

2. Closing-in (CI) subscale (item 15).

The correlations among the psychometric scales (PANSS, YMRS and the MADRS) and individual items and subscales of the SCPT revealed some very interesting points (Table 4). The PANSS-Positive subscale correlates inversely with the DfI and Cil. The PANSS-Negative subscale also correlates inversely with most indices. PANSS-General Psychopathology correlates again inversely with the DfI and Cil. The YMRS does not correlate with any index, and in the current study it was used in order to have a measure to compare with bipolar patients in future studies. The MADRS correlated negatively with most indices. From the above it is obvious that the relationship of schizophrenia and its psychometric profile to the cognitive function as assessed by the SCPT is rather complex and non-linear, and further research is necessary to uncover specific issues and mechanisms.

We believe that future factor analysis with the inclusion of different patient groups will help to further elucidate the mechanism underlying the performance in the SCPT.

## Conclusions

In summary, the current study has developed a reliable and valid instrument. The great advantage of this instrument is the fact that it is paper and pencil, easily administered and little time consuming and appropriate for use in non-organic mental patients. Further research is necessary to test its usefulness and its applications as a neuropsychological test.

## Competing interests

The authors declare that they have no competing interests.

## Authors' contributions

Konstantinos N Fountoulakis designed the study, analyzed the data, interpreted the results, wrote the draft and subsequent versions and finalized the manuscript

Melina Siamouli collected data, assisted in the interpretation of results, gave input to revisions of the manuscript and approved the final version

Panagiotis T Panagiotidis collected data, assisted in the interpretation of results, gave input to revisions of the manuscript and approved the final version

Stamatia Magiria collected data, assisted in the interpretation of results, gave input to revisions of the manuscript and approved the final version

Sotiris Kantartzis collected data, assisted in the interpretation of results, gave input to revisions of the manuscript and approved the final version

Vassiliki A Terzoglou collected data, assisted in the interpretation of results, gave input to revisions of the manuscript and approved the final version

Timucin Oral collected data, assisted in the interpretation of results, gave input to revisions of the manuscript and approved the final version

## Supplementary Material

Additional file 1 Standardised Copy of the Pentagons Test (SCPT). Click here for file
