# Supplementary material for: The standardised copy of pentagons test
Source: Ann Gen Psychiatry. 2011 Apr 11;10:13. doi: 10.1186/1744-859X-10-13 (PMC3080354; doi:10.1186/1744-859X-10-13)
Supplement: Additional file 1 — Standardised Copy of the Pentagons Test (SCPT). [file 1744-859X-10-13-S1.DOC]

**Additional file 1**

**Standardized Copy of the Pentagons Test (SCPT)**

***Fountoulakis et al. 2011***

| **Template:** | ***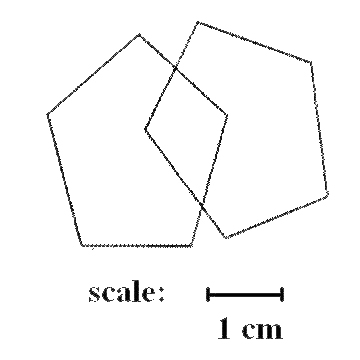*** |
| --- | --- |

# Instruction:

# Please copy the above drawing making a perfect identical one

#

|  |  | N of missing elements | Score |
| --- | --- | --- | --- |
| 1 | Number of left pentagon angles missing (max 5)Scoring Instructions:If the complete pentagon is missing record all 5 missing | 0  >0 | 100  0 |
| 2 | Number of right pentagon angles missing (max 5)Scoring Instructions:If the complete pentagon is missing record all 5 missing | 0  >0 | 100  1 |
| 3 | Number of angles of the overlapping shape (rhombus) missing or in excessScoring Instructions:Give 4 points if the rhombus is missing and count additional corners in case the overlapping shape is different | 0  >0 | 100  1 |
| 4 | Numbers of breaks and corrections in the lines of the two pentagonsScoring Instructions: | 0  1  2  3  4  5  >5 | 100  75  35  20  15  10  1 |
| 5 | Give 1 point for each severe distortion in the proportions in the left pentagon shapeScoring Instructions:Do not take into consideration missing angles and sides but only proportions | 0  >0 | 100  20 |
| 6 | Give 1 point for each severe distortion in the proportions in the right pentagon shapeScoring Instructions:Do not take into consideration missing angles and sides but only proportions | 0  >0 | 100  30 |
| 7 | Give 1 point for each severe distortion in the proportions in rhombus spapeScoring Instructions:Do not take into consideration missing angles and sides but only proportions | 0  >0 | 100  35 |
| 8 | Give 1 point for each angle with a reverse orientationScoring Instructions: | 0  >0 | 100  5 |
| 9 | Give 1 point if either of the pentagons (or the shapes if the design is poor) are markedly smaller (>30%) in comparison to its counterpart.Scoring Instructions:In order to rate size. both dimensions (height and width) should differ. Otherwise score only distortion and rate only items #5. 6 and 7. In case the drawing is both smaller and distorted score all items # 5. 6. 7 and 9 | 0  >0 | 100  15 |
| 10 | Give 1 point if the complete drawing is markedly smaller (>30%) in comparison to the template. | 0  >0 | 100  20 |
| 11 | Give 1 point for each side which is not a straight line | 0  1  2  3  >3 | 100  60  35  10  1 |
| 12 | Give 1 point for each angle whose sides are not straight lines but rather curved ones. | 0  1  2  3  >3 | 100  30  15  6  1 |
| 13 | Give 1 point if the design is rotated. | no  yes | 100  3 |
| 14 | Give 1 point for each crossing of the sides of the same pentagonScoring instructionsSample of sides crossing: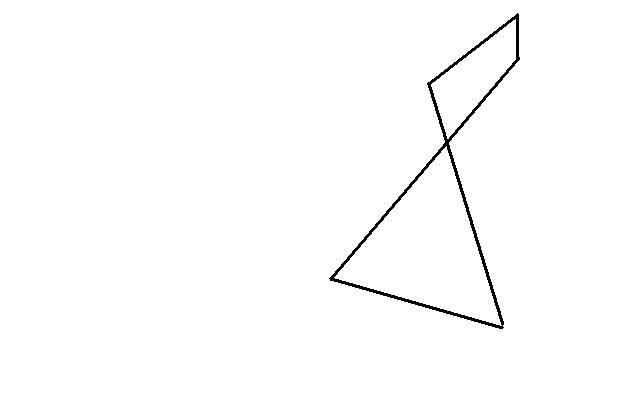 | 0  >0 | 100  0 |
| 15 | Give 1 point if the shape is closing-in | 0  >0 | 100  0 |
